# Inhibitory Activities of Alkyl Syringates and Related Compounds on Aflatoxin Production

**DOI:** 10.3390/toxins8060177

**Published:** 2016-06-07

**Authors:** Tomohiro Furukawa, Kurin Iimura, Taichi Kimura, Toshiyoshi Yamamoto, Shohei Sakuda

**Affiliations:** Department of Applied Biological Chemistry, The University of Tokyo, 1-1-1 Yayoi, Bunkyo-ku, Tokyo 113-8657, Japan; 9000163386@mail.ecc.u-tokyo.ac.jp (T.F.); akurin@mail.ecc.u-tokyo.ac.jp (K.I.); taichi-5909@ezweb.ne.jp (T.K.); a-yama@mail.ecc.u-tokyo.ac.jp (T.Y.)

**Keywords:** aflatoxin, inhibitor, alkyl syringate, alkyl paraben, alkyl gallate, respiration, complex II

## Abstract

Inhibitors of aflatoxin production of aflatoxigenic fungi are useful for preventing aflatoxin contamination in crops. As methyl syringate weakly inhibits aflatoxin production, aflatoxin production inhibitory activities of additional alkyl syringates with alkyl chains from ethyl to octyl were examined. Inhibitory activity toward aflatoxin production of *Aspergillus flavus* became stronger as the length of the alkyl chains on the esters became longer. Pentyl, hexyl, heptyl, and octyl syringates showed strong activity at 0.05 mM. Heptyl and octyl parabens, and octyl gallate also inhibited aflatoxin production as strongly as octyl syringate. Alkyl parabens and alkyl gallates inhibit the complex II activity of the mitochondrial respiration chain; thus, whether alkyl syringates inhibit complex II activity was examined. Inhibitory activities of alkyl syringates toward complex II also became stronger as the length of the alkyl chains increased. The complex II inhibitory activity of octyl syringate was comparable to that of octyl paraben and octyl gallate. These results suggest that alkyl syringates, alkyl parabens, and alkyl gallates, including commonly used food additives, are useful for aflatoxin control.

## 1. Introduction

Aflatoxin, a mycotoxin produced by some *Aspergillus* sp., is a potent, carcinogenic toxin that contaminates a wide variety of food and feed commodities, and thus is a serious problem worldwide [1,2,3]. However, it is difficult to resolve the problem due to the lack of an effective method to control aflatoxin production.

We have been studying aflatoxin production inhibitors, which do not inhibit the growth of aflatoxin-producing fungi, based on the idea that such inhibitors may be useful for prevention of aflatoxin contamination in food and feed without incurring a rapid spread of resistant strains [4]. In addition, highly selective aflatoxin production inhibitors are also useful as probes to investigate the basic regulatory mechanisms of aflatoxin production in fungi. To date, many compounds including plant constituents, pesticides, and microbial metabolites have been shown to be aflatoxin production inhibitors [4,5]. Recently, we found that respiration inhibitors, including commonly used pesticides, can also strongly inhibit aflatoxin production without significantly inhibiting the fungal growth [6].

**Figure 1:**
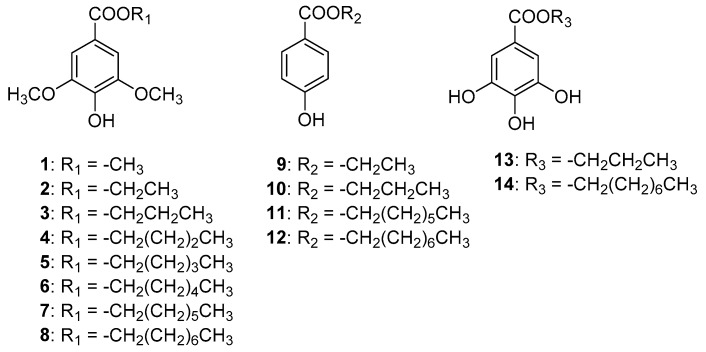
Structures of alkyl syringates (**1**–**8**), alkyl parabens (**9**–**12**), and alkyl gallates (**13**,**14**).

Methyl syringate (**1**, Figure 1) is an aflatoxin production inhibitor that was isolated from the essential oil of *Betula alba* [7]. Methyl syringate weakly inhibits aflatoxin production of *Aspergillus parasiticus* with high selectivity (half maximal inhibitory concentration (IC_50_) value of 0.9 mM). Our preliminary study on the structure-activity relationship of methyl syringate suggested that alkyl syringates with longer alkyl chains inhibited aflatoxin production more strongly than the original compound [4]. On the other hand, alkyl parabens and alkyl gallates, which include commonly used food additives, were shown to inhibit the complex II activity of the mitochondrial respiration chain [8,9]. The complex II inhibitory activity of five alkyl gallates with alkyl chains from pentyl to nonyl became stronger as the alkyl chain length became longer [8]. It was also known that complex II inhibitors such as siccanin, atpenin A5, mepronil, and boscalid inhibited aflatoxin production with selectivity [6]. These facts and the structural similarity of alkyl syringates to alkyl parabens and alkyl gallates may suggest that alkyl syringates inhibit complex II activity and, likewise, that alkyl parabens and alkyl gallates inhibit aflatoxin production through inhibition of mitochondrial complex II activity. In this paper, we report aflatoxin production and mitochondrial complex II inhibitory activities of alkyl syringates with alkyl chains from ethyl to octyl (**2**–**8**, Figure 1) and aflatoxin production inhibitory activities of alkyl parabens (**9**–**12**, Figure 1) and alkyl gallates (**13**,**14**, Figure 1).

## 2. Results and Discussion

### 2.1. Aflatoxin Production Inhibitory Activity of Alkyl Syringates and Related Compounds

**Figure 2:**
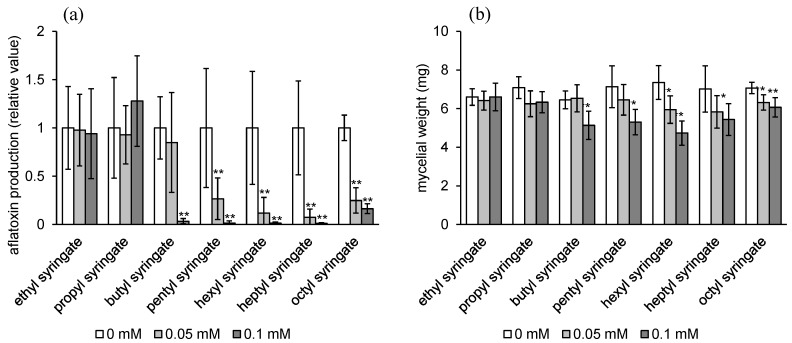
Effects of alkyl syringates (**1**–**8**) on aflatoxin production (**a**) and mycelial weight (**b**) of *A. flavus*. *n* = 6, ** *p* < 0.01, * *p* < 0.05, *vs.* control.

The inhibitory activities of alkyl syringates (**1**–**8**) on aflatoxin production of *Aspergillus flavus* IMF 47798 were examined at the concentrations of 0, 0.05, and 0.1 mM in a liquid culture. After four days of cultivation, the amount of aflatoxin involved in the culture supernatant and the fungal mycelial weight were measured (Figure 2). Methyl, ethyl, and propyl syringates (**1**–**3**) did not inhibit aflatoxin production at 0.1 mM. Butyl syringate (**4**) showed strong inhibitory activity at 0.1 mM. Pentyl, hexyl, heptyl, and octyl syringates (**5**–**8**) inhibited aflatoxin production very strongly at 0.05 mM. The IC50 value required for methyl syringate to inhibit aflatoxin production of *A. flavus* was 0.8 mM [7]; therefore, the aflatoxin production inhibitory activities of compounds **5**–**8** increase more than 20 times over the activity of methyl syringate (**1**). Five compounds (**4**–**8**) significantly reduced the fungal mycelial weight by, at most, around 30% of the control at the concentration of 0.05 or 0.1 mM (Figure 2b), but their strong inhibitory activity on aflatoxin production at the same concentration (Figure 2a) indicated that they inhibited aflatoxin production with relatively high selectivity. The strong aflatoxin production inhibitory activity of octyl syringate (**8**) was not changed after five and six days of cultivation without changing the fungal mycelial weight (Figure S1), suggesting that its effect on aflatoxin production is maintained for a long time.

**Figure 3:**
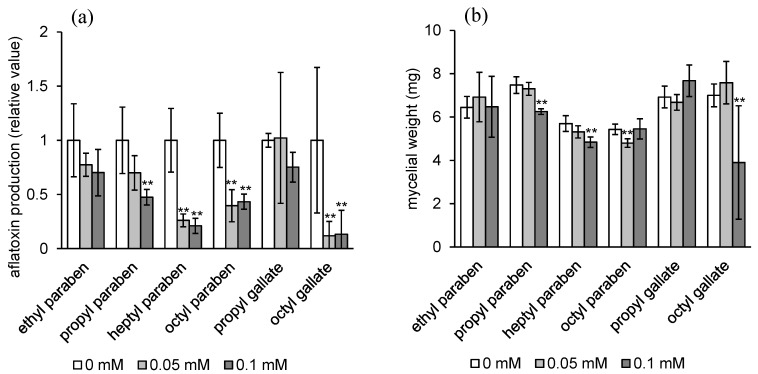
Effects of alkyl parabens (**9**–**12**), and alkyl gallates (**13**,**14**) on aflatoxin production (**a**) and mycelial weight (**b**) of *A. flavus*. *n* = 4–6, ** *p* < 0.01, * *p* < 0.05, *vs.* control.

The inhibitory activities of four alkyl parabens (**9**–**12**) and two alkyl gallate (**13**,**14**) on aflatoxin production of *A. flavus* were also examined (Figure 3). Aflatoxin production inhibitory activity of ethyl paraben (**9**) was weak. Propyl paraben (**10**) inhibited aflatoxin production more strongly than propyl syringate (**3**) and propyl gallate (**15**), but the inhibitory activity of propyl paraben was much weaker than that of heptyl paraben (**11**) and octyl paraben (**12**). Octyl gallate (**14**) inhibited aflatoxin production as strongly as octyl syringate (**8**) and octyl paraben (**12**) at the concentration of 0.05 mM. These strong aflatoxin production inhibitory activities of heptyl paraben (**11**) and octyl gallate (**14**) at the concentration of 0.05 mM were maintained after five and six days of cultivation, similarly to the case of octyl syringate (**8**) (Figure S1). Although propyl paraben has been reported to inhibit aflatoxin production of *A. flavus* at a concentration of 1 mM on a solid culture [10], we first showed the strong aflatoxin production inhibitory activities of heptyl and octyl parabens and octyl gallate in addition to those of the alkyl syringates mentioned above. Heptyl paraben and octyl gallate are commonly used food additives in the USA, and thus their new use in aflatoxin control has potential for future development.

Alkyl parabens and alkyl gallates have antifungal activity. The minimum inhibitory concentration (MIC) value of octyl gallate against *A. flavus* ATCC 9170 has been reported to be 0.063 mM [11]. In this study, octyl gallate (**14**) reduced the mycelial weight of *A. flavus* IMF 47798 at the concentration of 0.1 mM. However, at a lower concentration of 0.05 mM, octyl gallate did not affect fungal growth, while aflatoxin production of the fungus was strongly inhibited (Figure 3). Heptyl and octyl parabens (**11**, **12**) did not affect fungal growth at the concentrations tested, which indicates that heptyl and octyl parabens inhibit aflatoxin production with high selectivity.

### 2.2. Inhibitory Activities of Alkyl Syringates on Mitochondrial Complex II

Eight alkyl syringates (**1**–**8**) and related compounds were assessed for inhibitory activities on mitochondrial complex II using a commercial assay kit that uses complex II activity (succinate dehydrogenase activity) in bovine mitochondria. The IC50 values obtained are listed in Table 1. Complex II inhibitory activities of alkyl syringates were stronger as the length of the alkyl chain increased. The complex II inhibitory activity of octyl syringate (**8**) was as strong as octyl paraben (**12**) and octyl gallate (**14**). The IC50 value of boscalid, a fungicide targeting complex II, was much lower than the values of other compounds listed in Table 1. Boscalid potently inhibits aflatoxin production with the IC50 value of <0.01 μM in a liquid culture [6]. These results indicate that the aflatoxin production inhibitory activities of the compounds in Figure 1 correlate to the complex II inhibitory activities in Table 1. However, it is not clear whether the inhibitory activities on complex II in aflatoxigenic fungi are parallel to the inhibitory activities on aflatoxin production because there is little information on the inhibitory activities of the compounds on fungal mitochondrial complex II.

### 2.3. Effects of Octyl Syringate, Octyl Paraben, and Octyl Gallate on the mRNA Levels of Genes Encoding Proteins Responsible for Aflatoxin Biosynthesis

The aflatoxin biosynthetic gene cluster in *A. parasiticus* and *A. flavus* contains genes that encode biosynthetic enzymes and a regulatory gene, *aflR*. AflR regulates the transcription of most of the genes that encode biosynthetic enzymes, such as PksA, which catalyzes an early step in the aflatoxin biosynthetic pathway [12]. Therefore, AflR expression is necessary to initiate aflatoxin biosynthesis. We have been doing experiments with these genes, *aflR* and *pksA,* to estimate whether the target of an aflatoxin production inhibitor is present in a pathway before or after the transcription of *aflR* [13]. At a concentration of 4 mM, methyl syringate (**1**) significantly reduces the mRNA levels of *aflR* and *pksA* and aflatoxin production is repressed [7].

To test the effects of three octyl esters on the mRNA levels of *aflR* and *pksA*, *A. flavus* was cultured with octyl syringate (**8**), octyl paraben (**12**), or octyl gallate (**14**) at the concentrations of 0.05 mM. Subsequently, *aflR* and *pksA* mRNA levels were examined by quantitative PCR. None of the three octyl esters affected *aflR* and *pksA* mRNA levels (Figure S2).

These results suggest that the key modes of action of octyl syringate and methyl syringate for the inhibition of aflatoxin production may be different. Aflatoxin production inhibitory activity of octyl syringate may be correlated to its mitochondrial complex II inhibitory activity, as mentioned above. As octyl syringate does not repress the *aflR* transcription at the concentrations tested, octyl syringate might alter the metabolic flow of key molecules, such as acetyl-CoA, important for aflatoxin production [14]. Further work to investigate the mode of action of methyl syringate for inhibition of aflatoxin production is necessary to totally clarify the action of alkyl syringates on aflatoxin production. We are now investigating the detailed modes of action of methyl syringate and the complex II inhibitors described in this paper as well as other respiration inhibitors for the inhibition of aflatoxin production.

## 3. Conclusions

This study clarified that alkyl syringates (alkyl chains from butyl to octyl) and alkyl parabens (alkyl chains of heptyl and octyl) as well as octyl gallate can inhibit aflatoxin production strongly with selectivity. These aflatoxin production inhibitors also inhibit mitochondrial complex II activity. These inhibitors, especially those commonly used in food additives, may be useful as aflatoxin control agents.

## 4. Experimental Section

### 4.1. Strains and Culture Conditions 

*Aspergillus flavus* IMF 47798 was used as a producer of aflatoxins B_1_ throughout the study. Aflatoxin B_1_ is the main aflatoxin produced by the strain. IMF 47798 was maintained on potato dextrose agar (Difco, Franklin Lakes, NJ, USA). A preserved glycerol stock of a spore suspension prepared from a week-old culture (stored at −80 °C) was used as the inoculum. The spore suspension was inoculated into potato dextrose liquid media in 12-well microplates (2 mL/well). All test compounds were dissolved in dimethyl sulfoxide and added to the wells (final concentration of dimethyl sulfoxide was 0.1% *v*/*v*). The plates were incubated undisturbed at 27.5 °C for four days. Propyl and heptyl parabens, syringic acid, *p*-hydroxybenzoic acid, and octyl gallate were purchased from Tokyo Chemical Industry, Co., Ltd., Tokyo, Japan. Ethyl paraben and boscalid were purchased from Wako Pure Chemical Industries, Ltd., Osaka, Japan.

### 4.2. Synthesis of Alkyl Syringates, and Octyl Paraben

Ethyl syringate (**2**), propyl syringate (**3**), butyl syringate (**4**), pentyl syringate (**5**), hexyl syringate (**6**), and heptyl syringate (**7**) were prepared from syringic acid and the corresponding alkanol in the presence of H2SO4 according to the method described previously [7]. **2**: ESI/TOFMS (negative) *m/z* 225 (M − H)^−^; δ_H_ (CDCl_3_, 500 MHz): 7.31 (2H, s), 5.88 (1H, s, OH), 4.35 (2H, q, *J* = 7 Hz), 3.93 (6H, s), 1.38 (3H, t, *J* = 7 Hz); δ_C_ (CDCl_3_, 125 MHz): 166.5, 146.7 (C-3, 5), 139.2, 121.6, 106.7 (C-2, 6), 61.1, 56.5(CH_3_ × 2), 14.5, 10.6. **3**: ESI/TOFMS (negative) *m/z* 239 (M − H)^−^; δ_H_ (CDCl_3_, 500 MHz): 7.31 (2H, s), 5.90 (1H, s, OH), 4.25 (2H, t, *J* = 7 Hz), 3.80 (6H, s), 1.78 (2H, m), 1.01 (3H, t, *J* = 7 Hz); δ_C_ (CDCl_3_, 125 MHz): 166.5, 146.7 (C-3, 5), 139.2, 121.6, 106.7 (C-2, 6), 66.6, 56.5(CH_3_ × 2), 22.3, 10.6. **4**: ESI/TOFMS (negative) *m/z* 253 (M − H)^−^; δ_H_ (CDCl_3_, 500 MHz): 7.31 (2H, s), 5.90 (1H, s, OH), 4.30 (2H, t, *J* = 7 Hz), 3.93 (6H, s), 1.74 (2H, m), 1.44 (2H, m), 0.97 (3H, t, *J* = 7 Hz); δ_C_ (CDCl_3_, 125 MHz): 166.5, 146.7 (C-3, 5), 139.2, 121.6, 106.7 (C-2, 6), 65.0, 56.5(CH_3_ × 2), 30.9, 19.4, 13.9. **5**: ESI/TOFMS (negative) *m/z* 267 (M − H)^−^; δ_H_ (CDCl_3_, 500 MHz): 7.31 (2H, s), 4.28 (2H, t, *J* = 7 Hz), 3.93 (6H, s), 1.76 (2H, m), 1.39 (4H, m), 0.92 (3H, br.t, *J* = 7 Hz); δ_C_ (CDCl_3_, 125 MHz): 166.5, 146.7 (C-3, 5), 139.2, 121.6, 106.7 (C-2, 6), 65.2, 56.5(CH_3_ × 2), 28.5, 28.3, 22.4, 14.1. **6**: ESI/TOFMS (negative) *m/z* 281 (M − H)^−^; δ_H_ (CDCl_3_, 500 MHz): 7.31 (2H, s), 4.28 (2H, t, *J* = 7 Hz), 3.93 (6H, s), 1.75 (2H, m), 1.42 (2H, m), 1.33 (4H, m), 0.90 (3H, br.t); δ_C_ (CDCl_3_, 125 MHz): 166.5, 146.7 (C-3, 5), 139.2, 121.6, 106.7 (C-2, 6), 65.3, 56.5(CH_3_ × 2), 31.5, 28.8, 25.8, 22.6, 14.1. **7**: ESI/TOFMS (negative) *m/z* 295 (M − H)^−^; δ_H_ (CDCl_3_, 500 MHz): 7.31 (2H, s), 4.28 (2H, t, *J* = 7 Hz), 3.93 (6H, s), 1.75 (2H, m), 1.26–1.44 (8H, m), 0.88 (3H, br.t, *J* = 7 Hz); δ_C_ (CDCl_3_, 125 MHz): 166.5, 146.7 (C-3, 5), 139.2, 121.6, 106.7 (C-2, 6), 65.3, 56.5(CH_3_ × 2), 31.8, 29.0, 28.8, 26.1, 22.7, 14.1.

A tetrahydrofran solution of DCC (*N*,*N*′-dicyclohexylcarbodiimide, 2165 mg) was dropped into a tetrahydrofuran solution of syringic acid (991 mg) and 1-octanol (651 mg) at room temperature, and the mixture was stirred for 4 h at room temperature. After filtering the reaction mixture, the filtrate was evaporated to remove tetrahydrofuran and dissolved in ethyl acetate. The ethyl acetate solution was washed with aqueous 5% Na_2_HCO_3_, dried with anhydrous Na_2_SO_4_, and evaporated to dryness. The residue was loaded on a silica gel column (Wakogel C-200 silica gel, Wako, Osaka, Japan) and eluted with hexane–ethyl acetate (4:1, *v*/*v*). Octyl syringate (**8**, 706 mg, 46%) was obtained by crystallization. Octyl paraben (**11**, 421 mg, 34%) was prepared similarly from *p*-hydroxybenzoic acid (691 mg) and 1-octanol (651 mg). **8**: ESI/TOFMS (negative) *m/z* 309 (M − H)^−^; δ_H_ (CDCl_3_, 500 MHz): 7.30 (2H, s), 4.28 (2H, t, *J* = 7 Hz), 3.92 (6H, s), 1.75 (2H, m), 1.22–1.45 (10H, m), 0.87 (3H, br.t, *J* = 7 Hz); δ_C_ (CDCl_3_, 125 MHz): 166.5, 146.7 (C-3, 5), 139.2, 121.6, 106.7 (C-2, 6), 65.3, 56.5(CH_3_ × 2), 31.9, 29.3, 29.3, 28.8, 26.1, 22.7, 14.2. **11**: ESI/TOFMS (negative) *m/z* 249 (M − H)^−^; δ_H_ (CDCl_3_, 500 MHz): 7.94 (2H, d, *J* = 8.5 Hz), 6.88 (2H, d, *J* = 8.5 Hz), 4.29 (2H, t, *J* = 7 Hz), 1.75 (2H, m), 1.22–1.45 (10H, m), 0.87 (3H, br.t, *J* = 7 Hz); δ_C_ (CDCl_3_, 125 MHz): 167.1, 160.3, 132.0 (C-2, 6), 122.7, 115.3 (C-3, 5), 65.2, 31.9, 29.3, 29.3, 28.8, 26.1, 22.7, 14.2.

### 4.3. Aflatoxin Analysis

After four days of incubation, the culture broth of *A. flavus* IMF 47798 from each well was centrifuged to obtain the mycelia and culture supernatant. The mycelia were washed two times with 1 mL of distilled water and collected by centrifugation in a 2.0 mL microtube. After freeze-drying the mycelia, the mycelial weight was calculated by subtracting the weight of an empty 2.0 mL microtube from the total weight. 

To analyze aflatoxin B_1_ concentration in the culture supernatant, 0.5 mL of the supernatant was extracted with 0.5 mL of chloroform twice, and subsequently the chloroform (1.0 mL in total) solution was distilled off by air-drying. The remaining residue was dissolved in 0.1 mL of a 90% aqueous acetonitrile solution. The dissolved mixture was subjected to reverse-phase HPLC on a 250 mm × 4.6 mm inner diameter Capcell pak C_18_ UG120 column (Shiseido, Tokyo, Japan) by an isocratic elution of acetonitrile:methanol:water (10:30:60) over 20 min at a flow rate of 1.0 mL with detection at 365 nm (retention time: 8.3 min).

### 4.4. Complex II Activity Analysis

The inhibitory effects of test compounds on bovine heart mitochondrial complex II were assessed using the MitoCheck Complex II Activity Assay Kit (Cayman Chemical, Ann Arbor, MI, USA), according to the manufacturer’s protocol. A dilution series of each compound was prepared, and the percent inhibition of complex II activity was determined for each concentration. The IC_50_ value for each compound was calculated using the slope in which percent inhibition was plotted as a function of the concentration of the compound.

### 4.5. RT-qPCR Analysis of the Genes Encoding Proteins Responsible for Aflatoxin Biosynthesis

The lyophilized mycelia were ground under liquid nitrogen using a mortar and pestle. Total RNA was extracted with TRIzol reagent (Thermo Fisher Scientific, Waltham, MA, USA) and purified with the PureLink RNA Mini Kit (Thermo Fisher Scientific), according to manufacturer’s protocol. The cDNA was prepared with the ReverTra Ace qPCR RT Master Mix (TOYOBO, Osaka, Japan), according to manufacturer’s protocol. The cDNA derived from 0.05 μg of total RNA was used as a qPCR template. qPCR was carried out using FastStart Universal SYBR Green Master (Rox) (Roche, Basel, Switzerland) in a final volume of 25 μL for each reaction and an ABI 7300 Real-Time PCR System (Thermo Fisher Scientific). Two-step PCR conditions were as follows: after an initial incubation at 95 °C for 10 min, 40 cycles of 95 °C for 15 s and 60 °C for 1 min were performed. The amount of *aflR* and *pksA* mRNA were normalized to the amount of β-*tubulin* mRNA (control gene) in each sample. PCR primers used for each gene were as follows: β-*tubulin* 5′-AGCTCTCCAACCCCTCTTACG-3′ and 5′-TGAGCTGACCGGGGAAACG-3′; *AflR* 5′-TCCGCCATCTTTTCTCATCA-3′ and 5′-CCGAATTCCGAATCGACTGTTA-3′; *pksA* 5′-TGCATGGCGATGTGGTAGTT-3′ and 5′-GTAAGGCCGCGGAAGAAAG-3′.

### 4.6. Data Analysis

*A. flavus* IMF 47798 was cultured in potato dextrose liquid medium in a well of microplate, with or without a sample, at 27.5 °C for four days. Amounts of aflatoxin B_1_ in the culture supernatant and mycelial weight were measured according to above described methods. This experiment was repeated five times (*n* = 5). The aflatoxin amounts of each well were normalized to those from a control well. The normalized values were used for statistical analysis. Data are presented as the mean ± standard deviation (SD). Differences between groups were assessed by Dunnett’s test. 

## Figures and Tables

**Table 1:** Inhibitory activities of alkyl syringates and related compounds on mitochondrial complex II.

Compound	IC_50_ (mM)
methyl syringate (**1**)	- *
ethyl syringate (**2**)	- *
propyl syringate (**3**)	>20
butyl syringate (**4**)	9.7
pentyl syringate (**5**)	2.8
hexyl syringate (**6**)	1.1
heptyl syringate (**7**)	1.3
octyl syringate (**8**)	0.34
propyl paraben (**10**)	>20
octyl paraben (**12**)	0.16
octyl gallate (**14**)	0.29
boscalid	0.019

* No inhibition at 20 mM.

## Supplementary Materials

The following are available online at www.mdpi.com/2072-6651/8/6/177/s1, Figure S1: Effects of 0.05 mM of octyl syringate (**8**), heptyl paraben (**11**) and octyl gallate (**14**) on aflatoxin production (**a**) and mycelial weight (**b**) of *A. flavus* after four, five, six days of cultivation. *n* = 4. Figure S2: Effects of 0.05 mM of octyl syringate (**8**), octyl paraben (**12**) and octyl gallate (**14**) on *aflR* (**a**) and *pksA* (**b**) mRNA level of *A. flavus*. *n* = 6.
