# Supplementary material for: Inhibitory Activities of Alkyl Syringates and Related Compounds on Aflatoxin Production
Source: Toxins (Basel). 2016 Jun 7;8(6):177. doi: 10.3390/toxins8060177 (PMC4926143; doi:10.3390/toxins8060177)
Supplement: Supplementary file 1 [file toxins-08-00177-s001.pdf]

Supplementary Materials: Inhibitory Activities of Alkyl Syringates and Related Compounds on Aflatoxin Production

Tomohiro Furukawa, Kurin Iimura, Taichi Kimura, Toshiyoshi Yamamoto and Shohei Sakuda

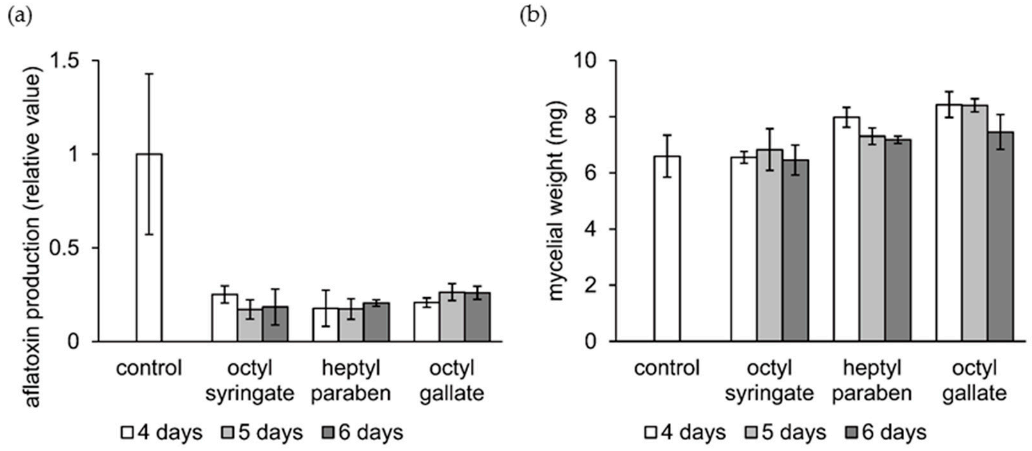

Figure S1. Effects of 0.05 mM of octyl syringate (**8**), heptyl paraben (**11**) and octyl gallate (**14**) on aflatoxin production (**a**) and mycelial weight (**b**) of *A. flavus* after four, five, six days of cultivation. $n = 4$.

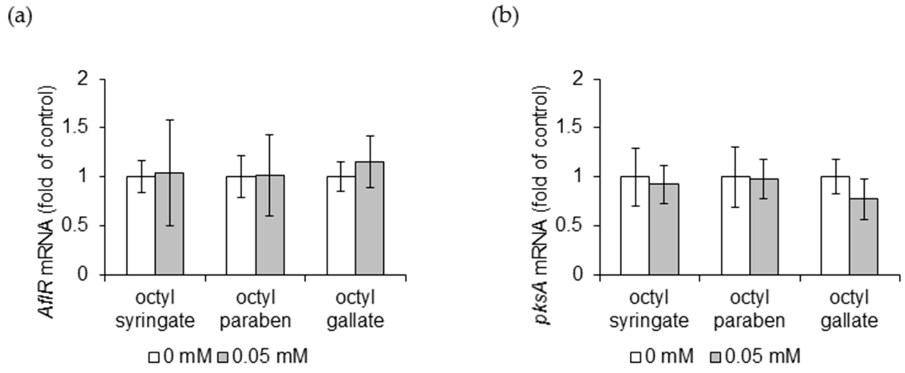

Figure S2. Effects of 0.05 mM of octyl syringate (**8**), octyl paraben (**12**) and octyl gallate (**14**) on *aflR* (**a**) and *pksA* (**b**) mRNA level of *A. flavus*. $n = 6$.
